# Migrant labour flows and interconnected agrarian transformations in Southern China

**DOI:** 10.1007/s10460-024-10692-y

**Published:** 2025-02-04

**Authors:** Yunan Xu, Chunyu Wang, Jingzhong Ye, Sai Sam Kham, Doi Ra, Jennifer C. Franco, Saturnino M. Borras

**Affiliations:** 1 Centre of Development Studies in the Department of Politics and International Studies (POLIS) of University of Cambridge, Cambridge, UK; 2 College of Humanities and Development Studies (COHD) of China, Agricultural University, Beijing, China; 3 International Institute of Social Studies (ISS) of Erasmus University Rotterdam, Rotterdam, Netherlands; 4 Transnational Institute (TNI), Amsterdam, Netherlands

**Keywords:** Agrarian transformation, Rural-urban migration, Cross-border migration, China, Myanmar

## Abstract

While contemporary debates about agrarian transformations that include topics like the persistence of family farms, agrarian capital accumulation, and simple reproduction squeeze remain vibrant, discussions about the increasingly significant role of migrant wage labour, which further complicates these processes, remain limited. In this paper, we argue that the process of capitalist accumulation in some sections of the agrarian and food system sectors in southern China is able to proceed despite recurring pressures — especially labour shortages in the rural economy caused by domestic rural–urban migration — because of the availability of migrant workers from neighbouring countries such as Myanmar. This development dynamic can be better understood through the lens of interrelated connections between land and labour and production and social reproduction across China and Myanmar. We highlighted the role of two land-related labour flows in the agrarian transformation in southern China–Myanmar regions. We argue that various social classes and groups across China and Myanmar are tied to land, wage work and capital accumulation and, in turn, their productive and social reproductive tasks are directly and indirectly linked to one another. If we are to understand agrarian transformation in settings like this, then methodologically, we will have to adopt an interconnected approach in order to bring the pieces of the puzzle together.

## Introduction

The dramatic transformation of the Chinese countryside and its contribution to the modernization of the Chinese economy is a key theme in research in Critical Agrarian Studies (Edelman and Wolford 2017; Akram-Lodhi et al. 2021; Borras 2023). Recent scholarship on agrarian transformation in China has been trailblazing. It is grounded in classical agrarian studies on social differentiation, capitalist accumulation, capital penetration into the countryside, rural-urban linkages, and land and labour transformation as productive forces in the rural economy develop^1^. Individually and collectively, these scholarly works have enabled us to better understand the logic that underpins the dramatic transformation of the Chinese countryside (Yan and Chen 2015; Huang, Yuan, and Peng 2012; Zhan 2019; Zhang 2015; van der Ploeg and Ye 2016). These relevant big debates revolve around classic themes about agriculture and family farms in China are all of great importance. However, these discussions tend to focus primarily on the land-labour dynamics internal to China. They can be further enhanced and broadened by including the ever-increasing China-global interconnections.

Specifically, the fragmented landscape in rural China caused by the rural land regime is closely associated with overseas land investments of varying types, ranging from formal, large-scale and corporate-dominated to informal, small-scale and individual investments (Hofman and Ho 2012; Bräutigam and Zhang 2013; Woods 2020). Moreover, dietary changes in China, particularly increased meat consumption, are linked with soaring soybean imports for animal feed (Oliveira and Schneider 2016; Lander et al. 2020). Such soy imports clearly affect the agrarian transformation in the soy-exporting countries in Latin America, but they also result in shifts in land use and agrarian structure in China, as Chinese farmers struggle to compete in the market with low-cost imported soy (Huang and Gao 2014; Yan, Chen, and Bun^2016^).

The situation in southern China is further complicated by the fact that there are two simultaneous land-related labour flows. On the one hand, the *outward flow* from rural southern China to urban centres across the country is in search of higher income through higher wages (Murphy 2002; Ye et al. 2013). According to the National Bureau of Statistics of China, there are approximately 300 million rural-urban migrants in China as of 2022. In 2022, the number of outward migration workers from Yunnan Province has exceeded 10 million.^2^ Such internal migration creates labour shortages for farmwork, affecting farmland that is still under households’ own operational control as well as lands they have leased to other small farmers or agrarian capitalists. On the other hand, there is an *inward flow* of migrant wage workers from Myanmar into farmlands in Yunnan, many of whom are farmers or pastoralists themselves with access to land in their home country, who come to Yunnan for mostly seasonal farmwork. This trend of cross-border migration partly filled the labour shortages in farming caused by internal rural-to-urban migration in China and shapes the farming practice and agrarian structure both in China and in the home countries of the migrants(Hua et al. 2019; Borras et al. 2022).

This reminds us that the character, pace and direction of agrarian transformations in China are conditioned, driven and affected not only by the processes and logic internal to China, but also by how China’s agrarian transformation is linked to dynamics of global capitalism. The latter manifests in many ways, including through cross-border flows of labour and capital. Empirically, in the agricultural and food systems sector of southern China, specifically Yunnan, the role played by migrant wage workers from Myanmar is huge, yet scholarly studies remain scant.^3^ Theoretically, the migration labour flows complicate the classic discussions of agrarian transformation around the persistence of farms and social differentiation (more elaboration in Sect. 2). This research focuses on the role that migrant wage work plays in agrarian transformation in southern China.

The period of the Covid-19 pandemic provided us with a window through which to focus on this topic. This paper is not about the pandemic; rather, the disruption in labour use and supply (the proximate cause of which was the pandemic) provides a lens through which we can see the vital role played by migrant wage workers. This paper employs a critical political economy lens and takes these interconnections into consideration. Our empirical data were collected through interviews and surveys in Yunnan and Myanmar across several years: 2017, 2019, 2021, and 2023.

Our argument is that the process of capitalist accumulation in the agrarian and food system sectors in southern China is able to proceed despite recurring pressures, especially labour shortages in the rural economy caused by domestic rural–urban migration, because of the availability of migrant workers from Myanmar. This development dynamic can be best understood from the perspective of interrelated connections between land and labour and production and social reproduction, both in China and in Myanmar. We hope this study makes a modest contribution to broader scholarly discussion on Chinese agrarian transformation more generally.

The remainder of the paper is organized as follows. We first illustrate the analytical framework of this study. Then, we introduce the data collection methods employed before, during and after the change in labour supply due to the global pandemic. The next section then offers an empirical analysis of the current land–labour nexus and social differentiation in rural China (particularly in rural Yunnan) and their interconnection with land-labour dynamics of Myanmar, including the cross-boundary land control in Myanmar and labour migration from Myanmar for sugarcane production. The subsequent section analyses the impact of the labour supply disruption on rural communities, which is both shaping and being shaped by the Chinese agrarian transformation in an interconnected way. The final section offers some concluding thoughts.

## Rethinking agrarian transformations

To understand the dynamics of the transformation of the Chinese countryside, this study uses a critical political economy lens that focuses on four fundamental questions in a dynamic and interrelated way: who owns what? (social relations of property); who does what? (dynamics of production); who gets what? (distribution of social, economic and environmental goods and bads); and what do they do with the created wealth? (dynamics of social and economic reproduction) (Bernstein 2010). Moreover, in our view, analysing land and labour within the production sphere, for example, farmland used to produce exchange value is important, but cannot on its own explain the dynamics of social change. Land and labour must be understood in the context of the “connected whole” of production/social reproduction, as Marx argued (Marx 1976, p. 711 [orig.1863]; see also Bhattacharya 2017; Fraser 2017; O’Laughlin 2022; Ossome 2022).

However, the agrarian political economy framework has various competing strands and traditions, with different perspectives in explaining the changes in smallholders and agriculture with the commodification process in the countryside. There are two main competing perspectives, including (a) social differentiation or polarization of middle farmers (a Leninist perspective) and (b) the persistence of middle family farms (a Chayanovian one) (Cousins 2023; van der Ploeg 2023). It is relevant to have a brief review of the key classic issues of contention about agrarian transformation.

Based on a study of Russia, Lenin concluded that as a result of capital accumulation, the middle peasants could completely get polarized into opposite groups, becoming either rural bourgeoisie (minority) with increasing capital control or the rural proletariat (majority) as the “double free labour” (free of land property and free to sell labour) (Lenin 1982, 130–137). Lenin argued that such social differentiation is a permanent, polarizing, economic process (Lenin 1982). In contrast to Lenin’s prediction about the demise of middle peasants, Chayanov believed that family farms would survive with the rise of capitalist relations. Compared with large-scale capitalist farms, family farms could hold greater competitive power through self-exploitation (van der Ploeg 2013, 16). Meanwhile, Chayanov further advocated for the combination of family farmers “within a self-governing cooperative structure” for the purposes of competing in the capitalist market (Shanin 2009, p. 98). In the same vein, Karl Kautsky argued that family farms could sustain and survive because of two advantages, namely, working longer and consuming less (Kautsky 1988, p. 110). For Kautsky, although small farms were much less efficient than large-scale capitalist farms, smallholders were able to share part of the benefits through “cooperative”. Under this cooperative arrangement, smallholders supplied household land and labour force to large capitalist farms in exchange for their access to credits, markets, and machines (Kautsky 1988, 120–126). Such “cooperatives”, according to Kautsky, were also indispensable for capitalist farms because this production form could supplement the large farms’ labour shortage and high input losses (Kautsky 1988, 147–166). Much of the contemporary debates are framed from these classic texts.

These debates around the differentiation and persistence of family farms are central to studies of contemporary agrarian transformation. However, the case of Yunnan province in China shows more dynamics that are not currently fully engaged in their entire complexities in agrarian scholarship on China. Many of the so-called farmers in parts of Yunnan are actually ‘peasant-workers’, that is, they are farmers in their formal hukou category in the sense that they are owners of farmland, which is operated by members of their households, but they are essentially workers in the cities (Andreas and Zhan 2016; Luo and Andreas 2020)^4^. They are able to retain their control over their land — and thus, persist as ‘smallholders’ — because they are able to tap into the flow of migrant wage workers from Myanmar. This differs from the typical understanding of the agrarian transformation process in both Lenin and Chayanov traditions. Because family farms are assumed not to engage, to any significant extent, in hiring in labour^5^. Meanwhile, the bigger capitalist entrepreneurs, who have managed to aggregate lands through leases and other means of land consolidation^6^, are able to expand largely because of the availability of wage workers from Myanmar. Each of these social groups is thus tied to land, wage work and capital accumulation and, in turn, their productive and social reproductive works are directly and indirectly linked to one another.

**Fig. 1:**
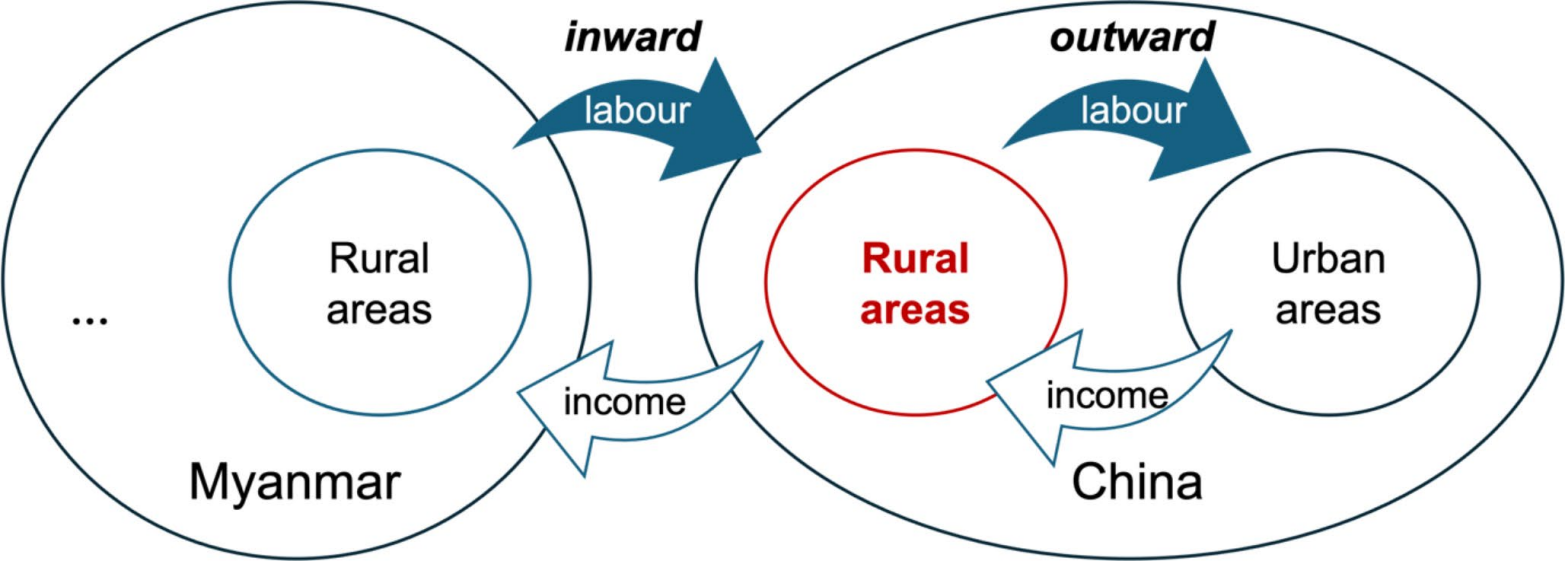
Interconnected agrarian transformation in China

Understanding these dynamics requires us to use an interconnected lens, considering the impacts of land-related labour flows, as shown in Fig. 1. On the one hand, there is a massive outward migration flow from rural southern China to urban centres across China. This outward labour flow is reflected in changes in the land and labour regimes in rural areas and is closely associated with the development of urban areas. First, such rural-to-urban migration is institutionally linked to China’s rural land regime, while the labour dynamics involved support China’s rapid domestic industrialization and urbanization via the massive supply of cheap labour(Wen 2021; Zhan 2019; Chuang 2020). Second, the left-behind population are mostly the elderly, children and those with health issues; many of them can oversee the continuous productive and social reproductive use of their household’s land, but are unable to work it by themselves and therefore leading to labour shortage in rural areas (Ye et al. 2013). Third, the rural labour changes resulting from large-scale internal migration, together with the changing food consumption patterns caused by the rise of the middle class in rapidly developing urban areas, lead to changes in land uses, land control and farming practices, with more chemical input-intensive production and higher-value agro-products, in what is described by Philip Huang as a “hidden agricultural revolution” (Huang 2016; see also Huang, Yuan, and Peng 2012). Fourth, the unequal access to incomes derived from internal migration further contributes to social differentiation in rural areas(Zhang 2015). Fifth, the outward migration flow generates wage incomes for rural households. These incomes are used to purchase production inputs (e.g., fertilisers or pesticides) and hire labour, thus allowing villagers to sustain their farming at home (van der Ploeg 2013; van der Ploeg and Ye^ 2016^).

On the other hand, the inward flow of cross-border migrant wage workers from Myanmar primarily fills the labour gap for agriculture in rural Yunnan (Hua et al. 2019). These cross-border migrant workers are relatively cheaper compared to local labour, making employment possible for both smallholders and large-scale producers in rural Yunnan. Thus, such inward and outward labour flows are essentially indispensable to understanding the persistence of family farms and the expansion of capitalist farms in Yunnan. Meanwhile, the cross-border migration also affects economic production and social reproduction in Myanmar. It essentially shapes the labour conditions and reproductive strategies within the households. Moreover, the wage incomes obtained by Myanmar migrant workers from farming in China not only sustain, and in a few cases even expand, the farming practices at their homes in Myanmar (since they can use the wage incomes earned in China to pay farmworkers at home to do some of the farming work while they are away), but also support part of the reproductive requirement of their households (Borras et al. 2022).

This reminds us that agrarian transformation nowadays cannot be understood in an isolated way but rather as an interconnected process. The transformation in one site is constantly (re)shaped by land and labour dynamics in adjacent or remote sites via massive land investments, trades and migration. This aligns with the concept of telecoupling in land use change politics(Eakin et al. 2014; Sun, Tong, and Liu 2017) and metacoupling in sustainable development(Liu 2023). Underlying this interconnected framework, this study will understand the agrarian transformation in rural Yunnan with a focus on the impacts of labour flow changes due to COVID.

### Method

The analyses in this paper largely rely on data collected from multiple fieldwork trips in Yunnan and Myanmar, and a household survey conducted in ZK County in Yunnan. These data were further supplemented by secondary sources collated via archive searches in international databases (e.g., FAOSTAT) and news websites.

Yunnan province, famous for its agricultural production, borders Myanmar, Laos and Vietnam, as shown in Fig. 1. The main cash crops in Yunnan include sugarcane, coffee, tea, fruits and tobacco (Pu, Huang, and Gao 2021). There is evidence of competition for land among the different crops, all of which also require a large amount of seasonal labour (Borras et al. 2018; Hua et al. 2019). These factors make Yunnan a suitable site for observing cross-border land and labour regime dynamics.

**Fig. 2:**
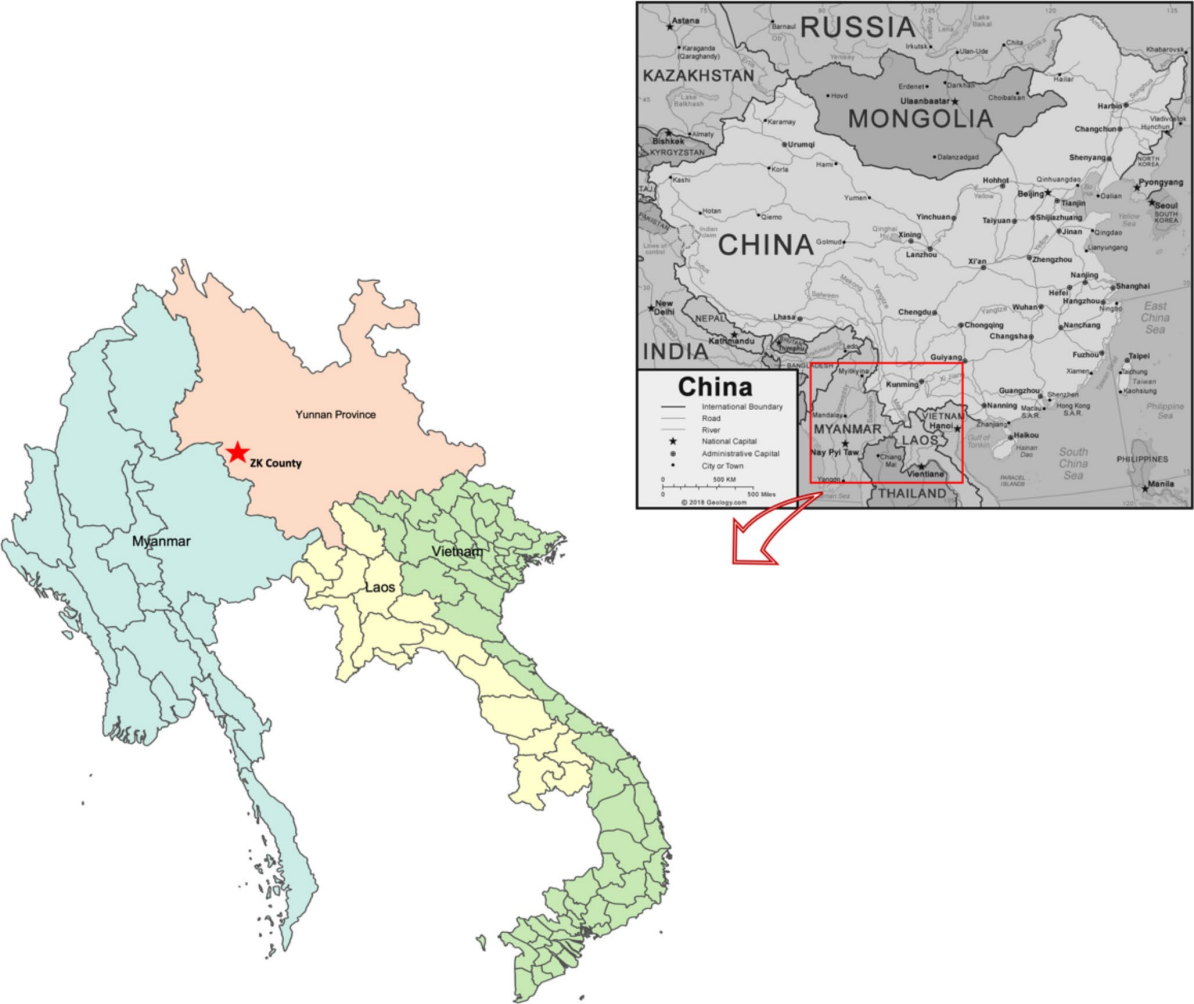
Map of ZK County

We picked ZK County, a boundary county (see Fig. 2), as our research site and focused on seasonal workers from Myanmar. Before and after the pandemic, the authors went to the field four times, together and separately. In the year 2017, we interviewed the sugar mill managers (two), seasonal Myanmar workers (fifteen), village leaders (four), large farmers (twelve), medium farmers (ten) and small farmers (eleven) in four different villages. Some of the authors also conducted fieldwork including interviews and focus group discussions inside Myanmar in 2019.

During the pandemic, the authors were able to carry out a survey in ZK County, Yunnan, to investigate the impacts of the disrupted migrant labour supply on villagers. In this survey, we deployed *Purposive sampling* (judgment sampling). This is a nonprobability sampling. Under this sampling, the participants are chosen deliberately based on the knowledge and experience of researchers and considering the willingness and availability of the participants (Etikan, Musa, and Alkassim 2015). Thus, this sampling technique can use limited resources to collect data efficiently. Also, it can include participants who hold different and important views or are situated in distinct and even extreme conditions(Campbell et al. 2020). Although this sampling method contains researchers’ bias and is “not good presentiveness of the population” (Etikan, Musa, and Alkassim 2015), this sampling method can better fit our research aim. Because for our research, instead of pursuing quantitative generalization or extending our conclusions to a wider region (larger population), we intend to explore the analytical generalization, this is, the social differentiation across the landscape and the distinct dynamics of these differentiated populations. By using purposive sampling, it offers us an opportunity to access those sub-groups, which have distinct social-economic features but are relatively small in size and thus are hard to be captured by random sampling (Klar and Leeper 2019). Specifically, we aim to collect data from households that either employ or do not employ migrant labour across varying scales of landholdings to explore how differentiated households are affected by the change of labour supply. In addition, this sampling technique also enables us to efficiently collect data within a short time frame, despite the challenges posed by the pandemic.

In this survey in ZK county, we paid particular attention to the size of landholdings, crop types and labour resources of villagers. ZK County had a total population of 176,356 in the year 2020, with seven towns and townships. As shown in Table 1, these towns and townships have varying degrees of reliance on migrant workers from Myanmar. This is related to the distinct labour needs for different key crops prevalent in the different towns or townships and specific local geographical conditions (e.g., land size, land quality and distance from the border). We conducted the survey in all seven towns and townships in 2021 and collected data from 176 households (incomplete questionnaires were excluded from the analysis). We have selected five main types of households, including (i) different land conditions (e.g. land abundant households and landless households), (ii) different land control (e.g. households that rent-out land and households that rent- in land), (iii) different crop choices (e.g. households that mainly plant sugarcane, households that mainly plant coffee, households that mainly plant tobacco, households that mainly plant banana, and households that mainly plant rubber, etc.), and (iv) different labour conditions (households that are mainly dependent on family labour, households that have extra labour power to do paid on-farm work locally, and households that are mainly dependent on employed local labour/labour from other provinces/ migrant labour from Myanmar).

**Table 1:** Questionnaires distributed and returned, 2021, ZK County

Towns/Townships	Population ^a^	Percentage share of seasonal Myanmar workers in labour use ^b^	Number of questionnairs distributed	Valid questionnaires returned
Pheonix Town	18,166	Seasonal Myanmar workers accounted for approximately 18.67% of the total agricultural workers. The figurue dropped to 7.1% due to the pandemic in 2020.	25	20
Umbrella Town	40,417	Seasonal Myanmar workers were around 60% before the pandemic.	50	45
MP Town	43,097	Seasonal Myanmar workers usually accounted for 30% before the pandemic.	46	41
MB Township	22,178	Around 2–3% before the pandemic	31	26
MD Township	20,636	Around 12%, or over 1,000 people before the pandemic.	20	16
Forest Township	18,666	Around 1% before the pandemic	16	21
Army Township	13,196	Around 20% before the pandemic	12	7
**Total**	**176,356**	**-**	**200**	**176**

^a^https://www.hongheiku.com/xianjirank/ynsgxsq/8513.html, ^b^data collected in the online investigation in 2020

## Interconnection

The agrarian transformation in rural Yunnan, particularly the transition of land and labour regimes, is not occurring in isolation but is partly shaped by Myanmar’s transformation.^7^ The land and labour dynamics of the two regions are mutually embedded, and are necessarily multi-sited. There are two key interconnections, namely: (i) high transaction costs of land investments in China related to the rural land system which contribute to current waves of land investments via both formal and informal means for sugarcane production in Myanmar^8^; and (ii) the rural labour shortage caused by internal rural–urban migration in China which leads to massive cross-border labour migration from Myanmar. These dynamics in turn shape the land–labour nexus, production and social reproduction in both regions.

### Land dimension

Changing patterns in land use and land control among the rural households in China that are reference points for outward flows of household members and inwards flows of migrant wage workers can be seen and understood by looking at the institutional dynamics that historically shape the politics of land in China. In Yunnan, similar to other regions of China, rural farmland was distributed to rural households under the household responsibility system (HRS) reform in the early 1980s (Ye 2015). The initial land distribution was conducted under the principle of fairness (Unger 2002). Specifically, the land in each village was firstly classified into different types according to quality (usually three to five types). Each type of farmland was then divided and allocated to villagers according to the size of each household. Thus, each household usually got several tiny land plots of various quality and spatially dispersed. This contributed to what some observers call a highly fragmented rural landscape (Nguyen, Cheng, and Findlay 1996; Tan et al. 2006).^9^ In addition, land politics in boarder areas of Yunnan also shaped by small boarder politics (Sturgeon 2004, 2012).

Meanwhile, demand for oilseeds, animal feed and sugar sources has risen as a result of the change in diet patterns towards more oily and animal-sourced foods and sugar-sweetened beverages (Si and Scott 2019). This has led many investors, both corporations and individual entrepreneurs, to invest either in direct land acquisition in Myanmar or in importation of commodities produced in Myanmar. This is encapsulated in the recent wave of land investments (both formal and informal, large-scale and pin-prick type) in Myanmar for sugarcane production (see Table 2). In addition, the state-direct Opium Replacement Programme has also offered motivations for Chinese investors to invest in cross-border regions via subsidies (Lone and Cachia 2021; Lu and Dwyer 2023). These land investments are usually located near the border, as sugarcane is a crop that requires timely processing after being cut^10^. After harvesting, sugarcane is transported by trucks across the border for processing in sugar mills in Yunnan. These cross-border land investments are conducted by entrepreneurs and/or villagers and are mostly pin pricks, but do have a critical role in shaping the agrarian transformation in both China and Myanmar (Hua et al. 2019). One example is a villager from ZK County whom we interviewed in 2023. Between 2017 and 2020, he controlled 300 mu sugarcane plantations in Myanmar through informal land lease, facilitated by a village leader in Myanmar^11^. The cultivation of his sugarcane plantations in Myanmar largely relied on local labour, which is much cheaper compared to labour costs inside China. Then, he transported all the output produced in Myanmar to sugar mills in China. The villager described his practices as informal “individual endeavour”.

These Chinese land acquisitions in Myanmar are partly conditioned by the fragmented rural land system in Yunnan, but it is not an entirely one-way process; conditions inside Myanmar itself are also influential in facilitating such cross-border flows and shaping the trajectory of social change (Ra et al. 2021), altering the social relations around land control and land use in Myanmar in both intended and unintended ways. Moreover, flows of capital, land access and labour across the Myanmar–China border are not limited to the sugarcane sector; for cases involving banana, corn, rubber and nuts, for example, see Borras, Franco, and Nam (2020) and Ra (2022).

**Table 2:** Before, during and after the pandemic, the size of sugarcane plantations controlled by actors from ZK County in Myanmar

Crushing season	Total plantation area (mu)	Tons of cane crushed
2016/2017	N/A	223,045
2017/2018	N/A	317,883
2018/2019	N/A	352,969
2019/2020	80,100	391,157
2020/2021	83,000	319,561
2021/2022	69,900	160,991
2022/2023	50,000	92,093

Data source: provided by NH Sugar Mill Corporation and the Bureau of Agriculture and Rural Affairs, ZK County

**Fig. 3:**
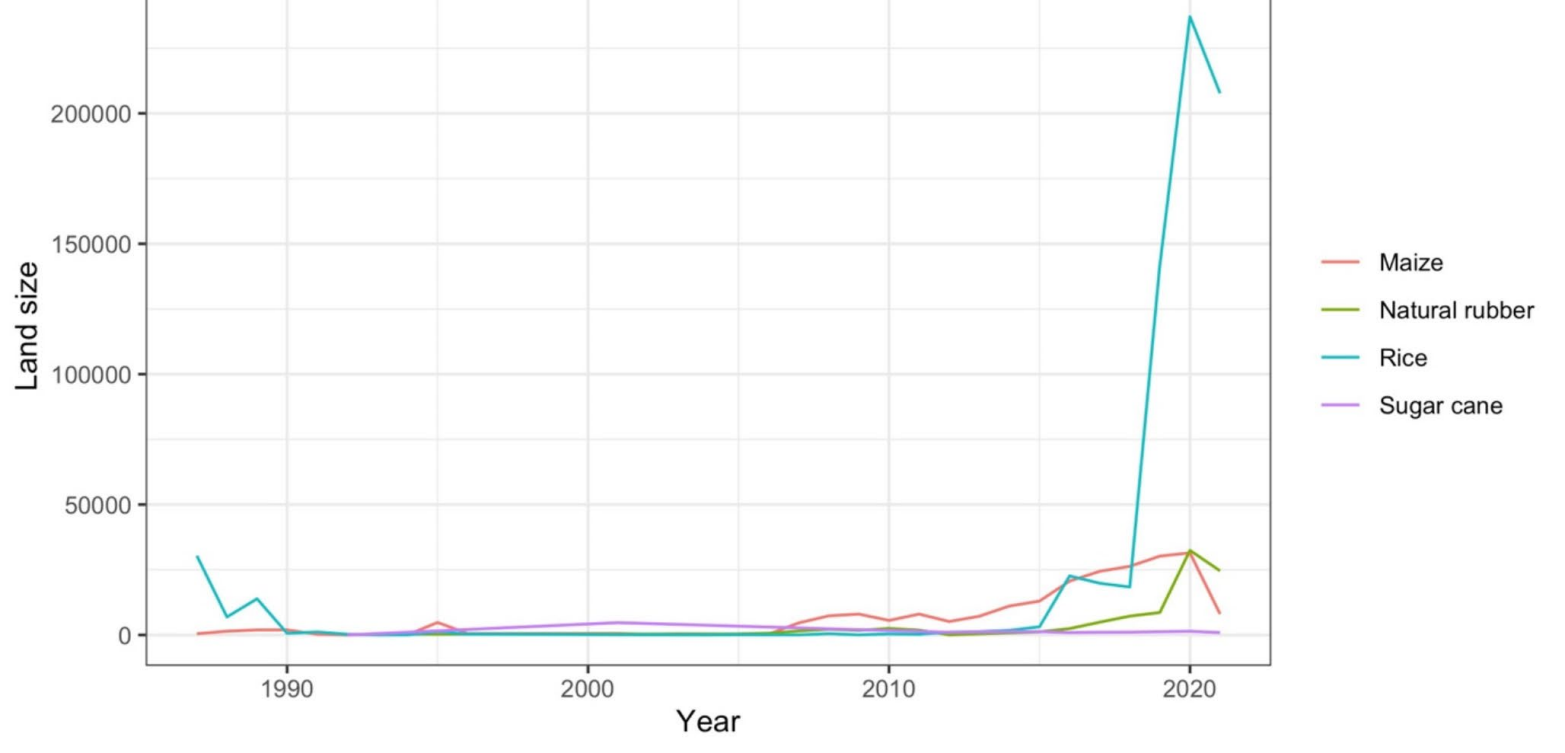
Indirect land control by China in Myanmar via trade of key crops. We take the four key crops into our calculation, including maize, rice, natural rubber and sugarcane. Bananas are a significant crop exported from Myanmar to China; according to FAOSTAT, 12,700 tonnes of bananas were exported to China in 2022. However, due to the lack of annual yield data for bananas, it is not possible to estimate the indirect impact caused by banana exports.

In addition to direct land access, China’s imports of cash crops produced from land across the border indirectly shape the dynamics of land use and land control in the exporting countries. This agro-product trade essentially means that China acquires an indirect but commanding control over land, *as argued by Xu and* Borras (2024). To better understand the scale of such indirect land control, we convert the quantity of key cash crops traded to China to an equivalent land area. We estimate that in 2020, China had indirect control over approximately 302,000 ha of rural land in Myanmar (see Fig. 3). This process has significantly affected the land use and control and labour dynamics in Myanmar (Borras et al. 2022). However, gaining a deeper understanding of these effects will require further research.

The calculation is based on the following equation.

$$ AIP = C_{P} \sum_{{h = 1}}^{n} \frac{{I_{{h,j}} \times 10000}}{{Y_{{h,j}} }} $$

Here *AIP* is the land area of imported agro-product P, and the unit is ha. *C*_*p*_ is the conversion factor of a certain agro-product p. For primary products, the conversion factor is 1. *I*_*h, J*_ is the import quantity of agro-product p from a country h in the year j. These data on import quantities are accessed from FAOSTAT. The unit is tonnes. *Y*_*h, J*_ is the yield of agro-product p from a country h in the year j. These data on import quantities are accessed from FAOSTAT. The unit is hg/ha.

### Labour dimension

**Fig. 4:**
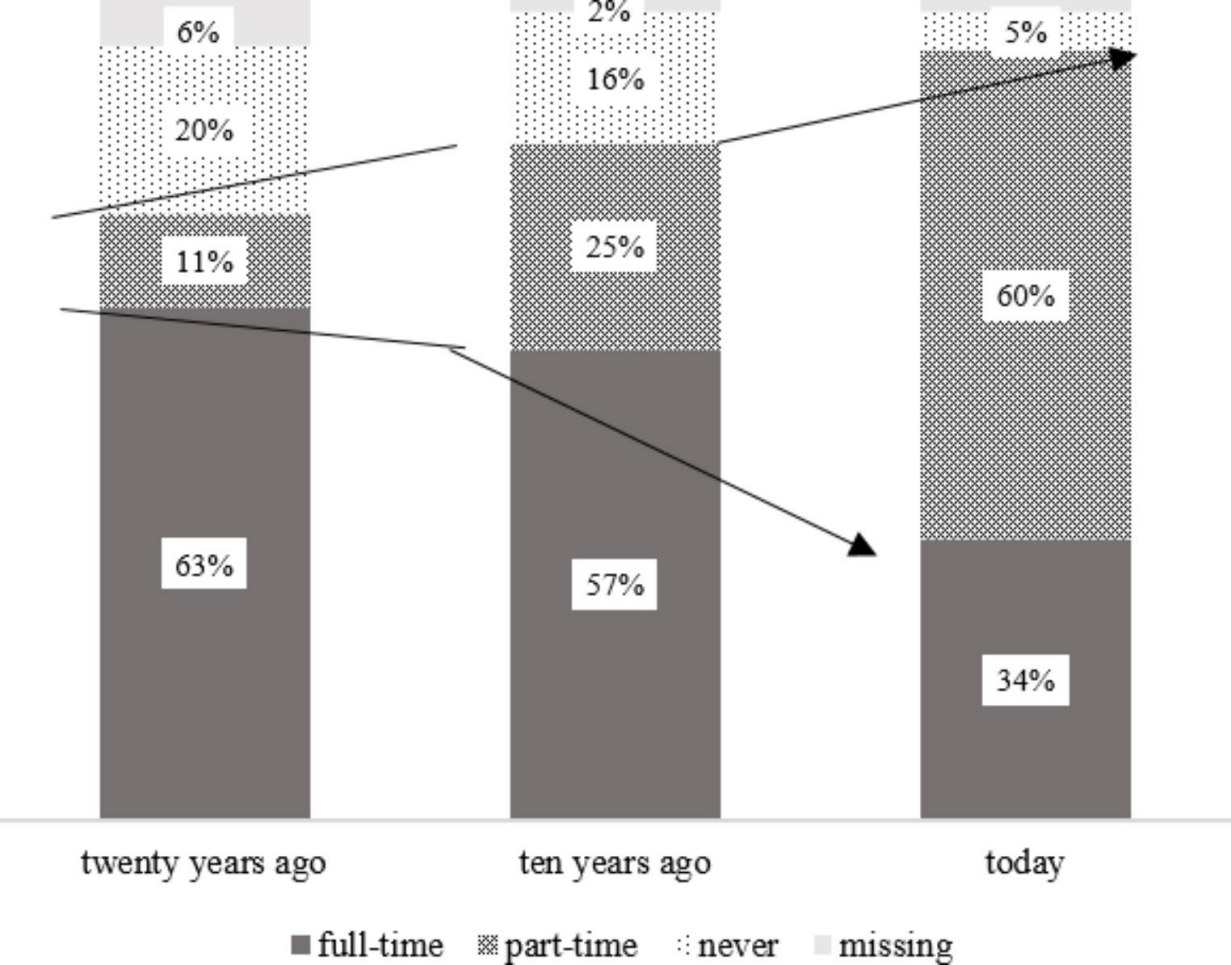
Shift of farming time in rural Yunnan. Data source: a survey in ZK county in Yunnan province in August 2021.

**Fig. 5:**
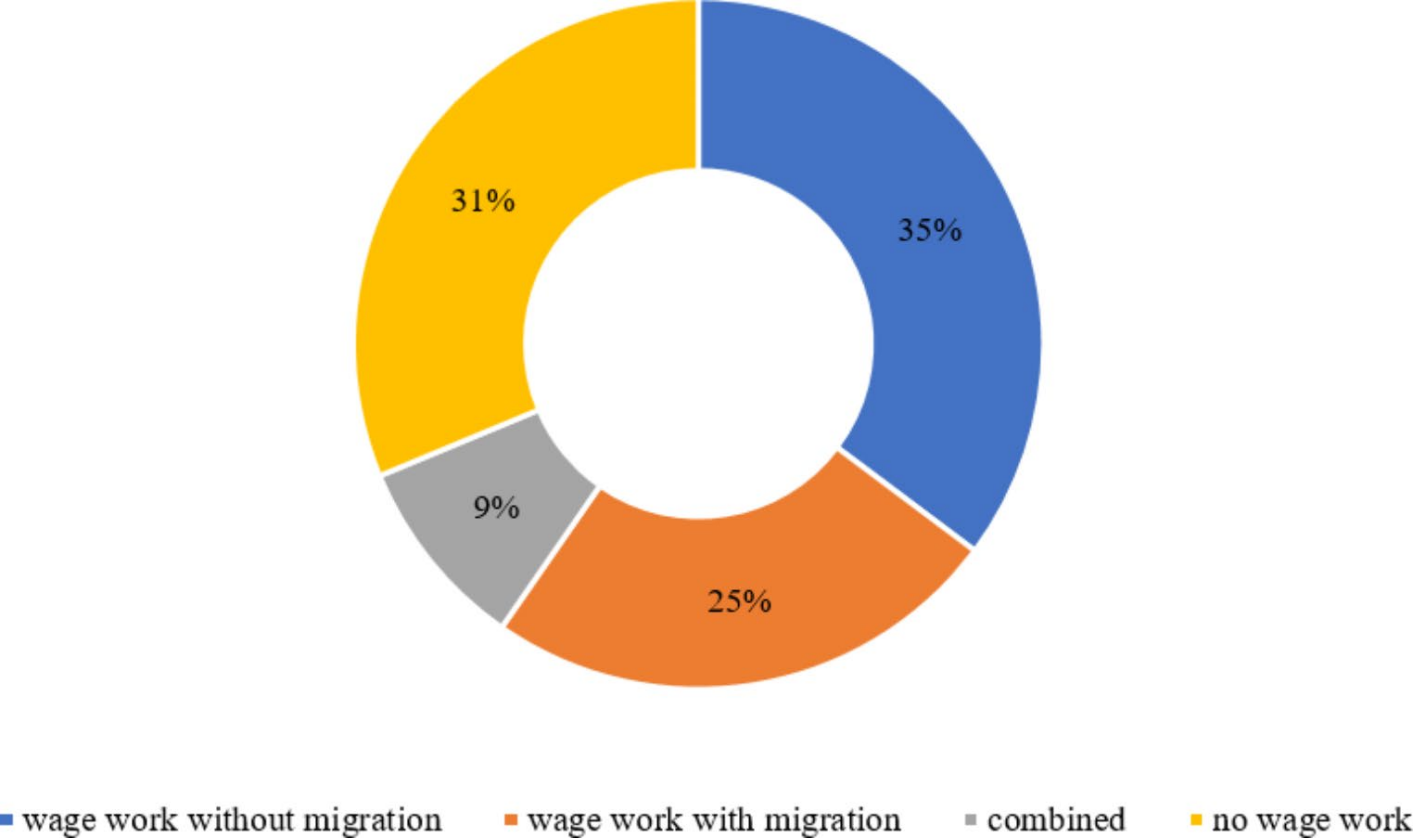
Wage work status among the surveyed households(*N* = 176). Data Source: survey in ZK county in Yunnan province in August 2021.

Regarding labour conditions, there is a trend of livelihood diversification in rural Yunnan as shown in Fig. 4. Over the past twenty years, the proportion of rural households engaging in full-time farming has declined from 63 to 34%. Meanwhile, the percentage of rural households that conduct part-time farming has risen sharply from 11 to 60%. This shift is related to the prioritization of off-farm endeavours in rural China in response to the country’s rapid urbanization and industrialization. Many villagers in rural Yunnan choose to undertake wage work either in the nearby towns or further away. As shown in Fig. 5, most rural households surveyed (69%) are doing wage work, with or without migrating.^12^

This implies a huge labour shortage for farming, particularly for the cultivation of labour-intensive crops such as sugarcane. To fill the labour gap, rural households started to employ labour externally (Wang and Xu 2022). With the rise in labour costs inside China, cheap migrant workers from Myanmar have tended to become the dominant labour force employed. According to our fieldwork, “there are about 20,000 migrant workers coming from Myanmar to ZK County in the cutting season every year. They usually stay [in China] for four months” (fieldnotes, March 11, 2020). In Pheonix Township, the number of migrant workers from Myanmar usually reaches more than 1,660, accounting for 18.67% of the total rural labour force. In Mengpeng Township, where there are large areas of sugarcane plantation, migrant workers usually account for 30% of the total rural labour force (Online interview, March 5, 2021). In Bangdong Village, there were around 300 sugarcane cutters from Myanmar in 2019; they usually come in January and return home for the Water-Sprinkling Festival () in April. Before workers from Myanmar first appeared in Bangdong, the village organized mutual aid teams to harvest the sugarcane, but this became increasingly difficult because so many villagers migrated out to work and the sugarcane plantation area kept expanding. The village stopped organizing mutual aid teams in 2014 (fieldnotes, April 21, 2019).

This phenomenon of migrant labour seems an anomaly at first glance: small or medium-sized producers are observed to employ labour for farming, which differs from the typical understanding of peasants’ way of farming (van der Ploeg 2013). However, it actually reflects the impacts of migration flows on the agrarian transformation. Most villagers in rural Yunnan retain control of land in their home village as a safety net. They can earn an average of 100–120 yuan per day as a wage worker in town, and 200–300 yuan per day when undertaking wage work in a big city in Guangdong. Meanwhile, employing a seasonal worker from Myanmar for sugarcane cutting costs about 70–80 yuan a day. The wage income earned by villagers from off-farm work thus sustains their farming at home because it enables them to employ migrant workers from Myanmar.

Such labour dynamics also have a significant impact on social reproduction in China, bearing in mind that economic production and social reproduction are parts of a connected whole (Marx 1976 [orig.1863]; Cousins 2022). On the one hand, wage income is a vital part of rural household income needed to maintain and reproduce the labour force, including access to education and healthcare services. On the other hand, the trans-local dynamics of rural households are closely related to shifts in rural households’ strategies to meet productive needs, including the intra-household division of labour, and gender and intergenerational relationships (Ye et al. 2016; Jacka 2018).

**Fig. 6:**
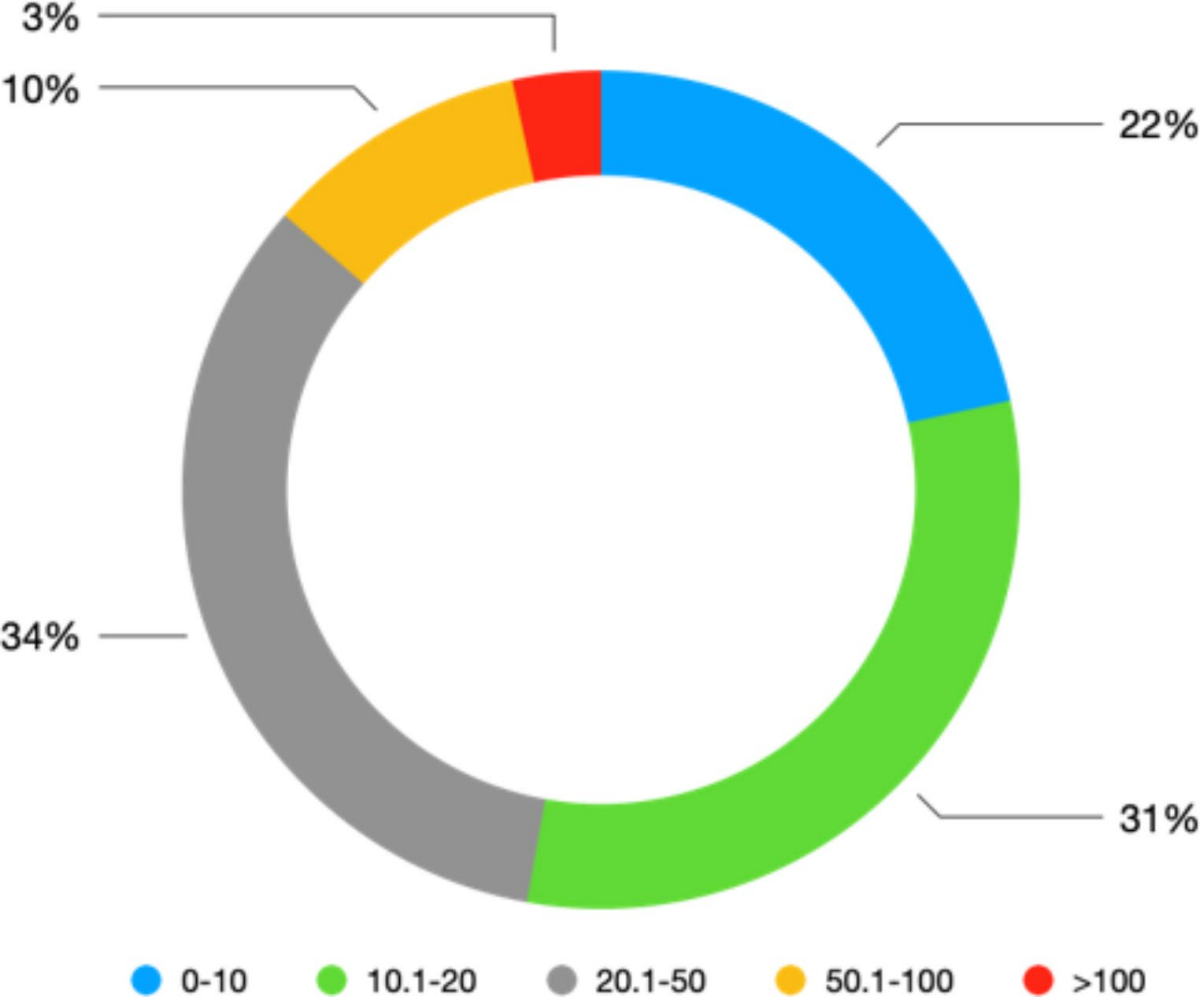
Land access of rural households in Yunnan (*N* = 176, unit: mu). Data Source: survey in ZK county in Yunnan province in August 2021.

As shown in Fig. 6, villagers in rural Yunnan are socially differentiated in terms of land access, although the majority of the villagers (around 87%) still access less than 50 mu. The difference in access is related to the ability of a few villagers to accumulate land via both economic (e.g., land leasing) and extra-economic ways (e.g., land reclaim and land enclosure with their advantageous access to labour and social capital)^13^. This ability of the few is closely related to villagers’ unequal access to wage work opportunities, social capital and family labour resources (Huang, Yuan, and Peng 2012; Yan and Chen 2015; Zhang 2015).

There is also a close association between differentiated land access and the labour arrangements of rural households. Table 3 shows that the average size of farmland for households not hiring labour is 26.3 mu, while for those hiring labour it is 45.3 mu. The significance of the differences is determined by the independent t-test. As shown in this table, the difference between these two groups ( i.e. households that hire labour and that do not) is significant. This implies a close relationship between land size and labour employment: those rural households with access to larger tracts of farmland tend to hire external labour.

**Table 3:** T test result of land access between households who hire labour and who do not

	Mean	Median	Sig.
Hiring labour (*N* = 46)	45.29	30	0.005
Not hiring labour (*N* = 130)	26.32	20	

Data Source: survey in ZK county in Yunnan province in August 2021

When *P* < 0.05, the difference among the groups is significant. More statistical results on this T-test, see Table 4 in the Appendix

**Fig. 7:**
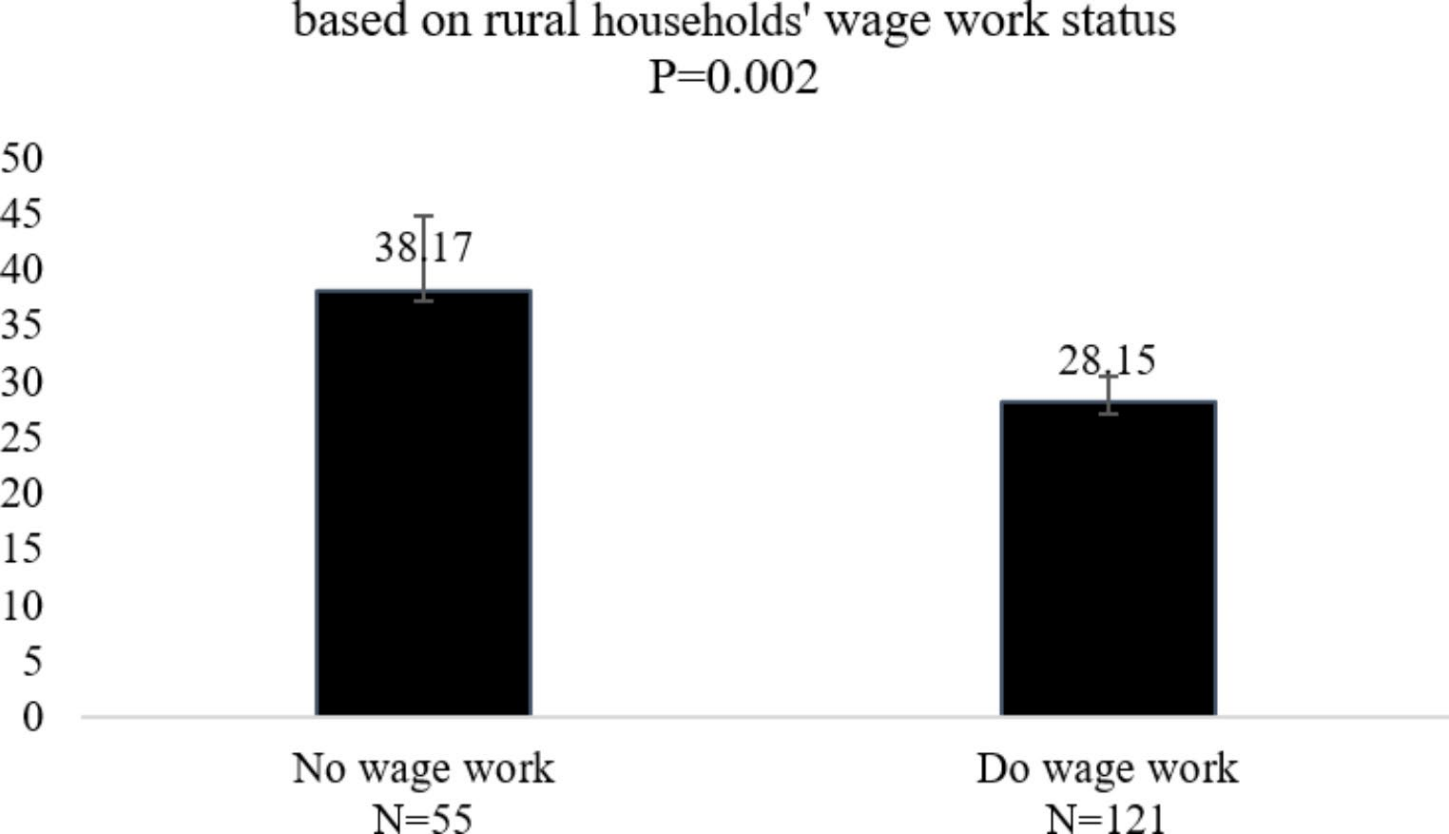
Differentiated farmland size controlled by villagers who do wage work and who do not (unit: mu). Data Source: survey in ZK county in Yunnan province in August 2021. The significance of the differences is determined by the independent t-test. When *P* < 0.05, the difference among the groups is significant. More statistical results on this indeppendent T-test, see Table 5 in the Appendix.

Meanwhile, as shown in Fig. 7, the average farmland size of villagers doing wage work is 28.1 mu, while that of those not doing wage work is 38.1 mu, and This means that rural households with members doing wage work tend to control a smaller amount of farmland.

These dynamics reflect mutually embedded land and labour relations between China (Yunnan) and neighbouring countries (Myanmar). Thus, the impacts of changes in labour use and supply on rural communities since the pandemic are necessarily shaped by and shaping agrarian transformation within and across countries.

## The impact of changes in labour supply on the Chinese agrarian transformation

The COVID-19 pandemic erupted at the end of 2019. To avoid the spread of the virus, a series of policies, including lockdowns (closed management of communities) and the closing of national borders between China and Myanmar, were launched.^14^ These measures did lead to changes in labour supply in rural China in two main ways.

**Fig. 8:**
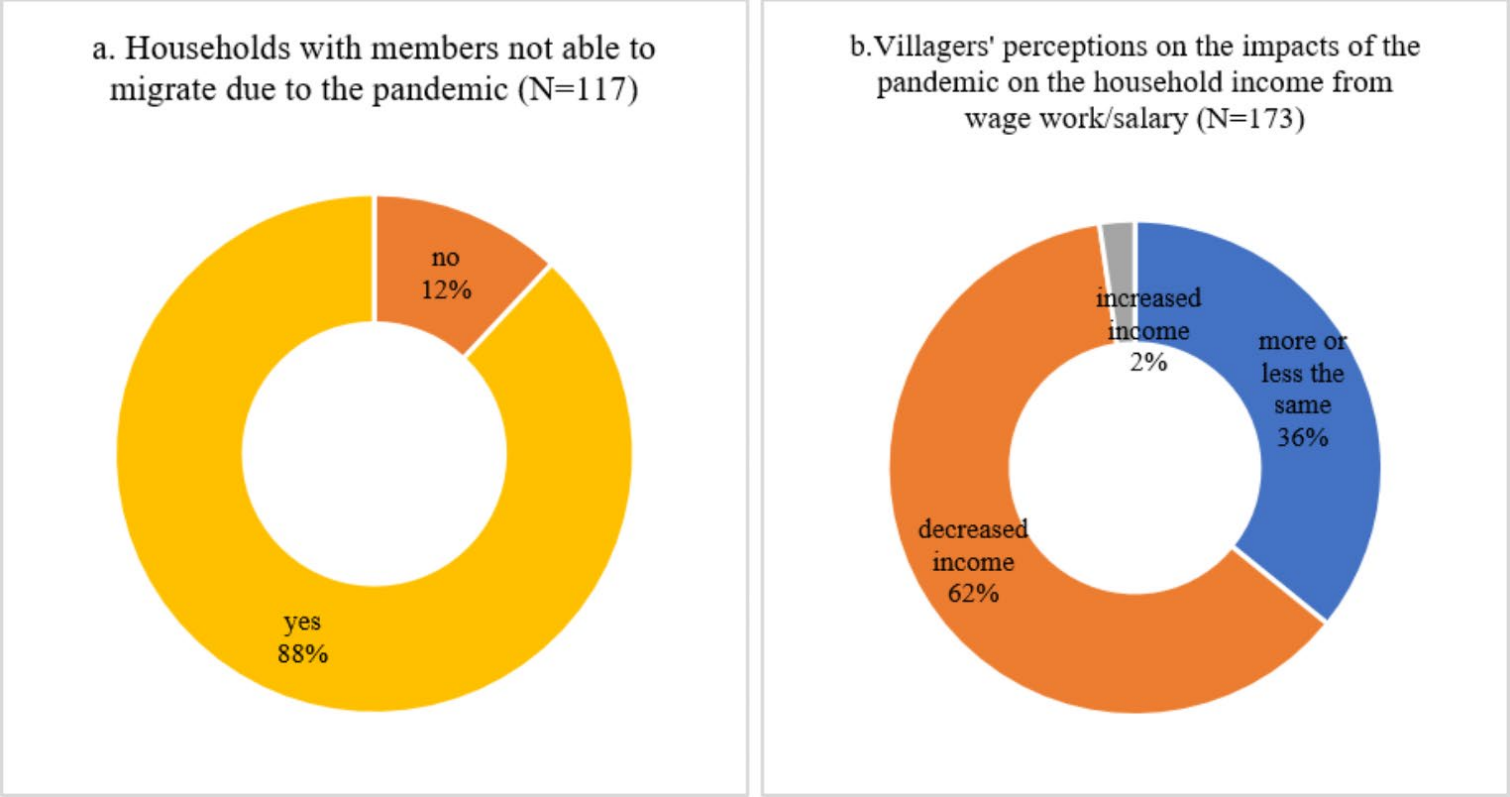
Impacts of the pandemic on wage work and migrations. Data source: survey in ZK county in Yunnan province in August 2021. Noted Only respondents who answered these questions were calculated

Firstly, villagers who used to migrate to undertake wage work in different urban centres of China were not able to travel to the urban areas during the pandemic. According to our survey in ZK County in Yunnan in 2021, around 88% of rural households had members who were unable to migrate to urban areas due to the impacts of the pandemic (see Fig. 8a). For small farms, these villagers who temporally could not migrate to urban areas partly filled the labour demand during the pandemic. But for large-scale producers, there is still a big gap without migrate workers from Myanmar.

Meanwhile, given the prevalence of wage work in rural China (as mentioned in the previous section), the inability to engage in wage work indicates a significant negative impact on rural households’ income. As shown in Fig. 8b, more than 62% of the villagers thought their wage work income had significantly decreased due to the pandemic. Such a decrease in household incomes tends to negatively affect these villagers’ economic production (e.g., farming) and social reproduction.

**Fig. 9:**
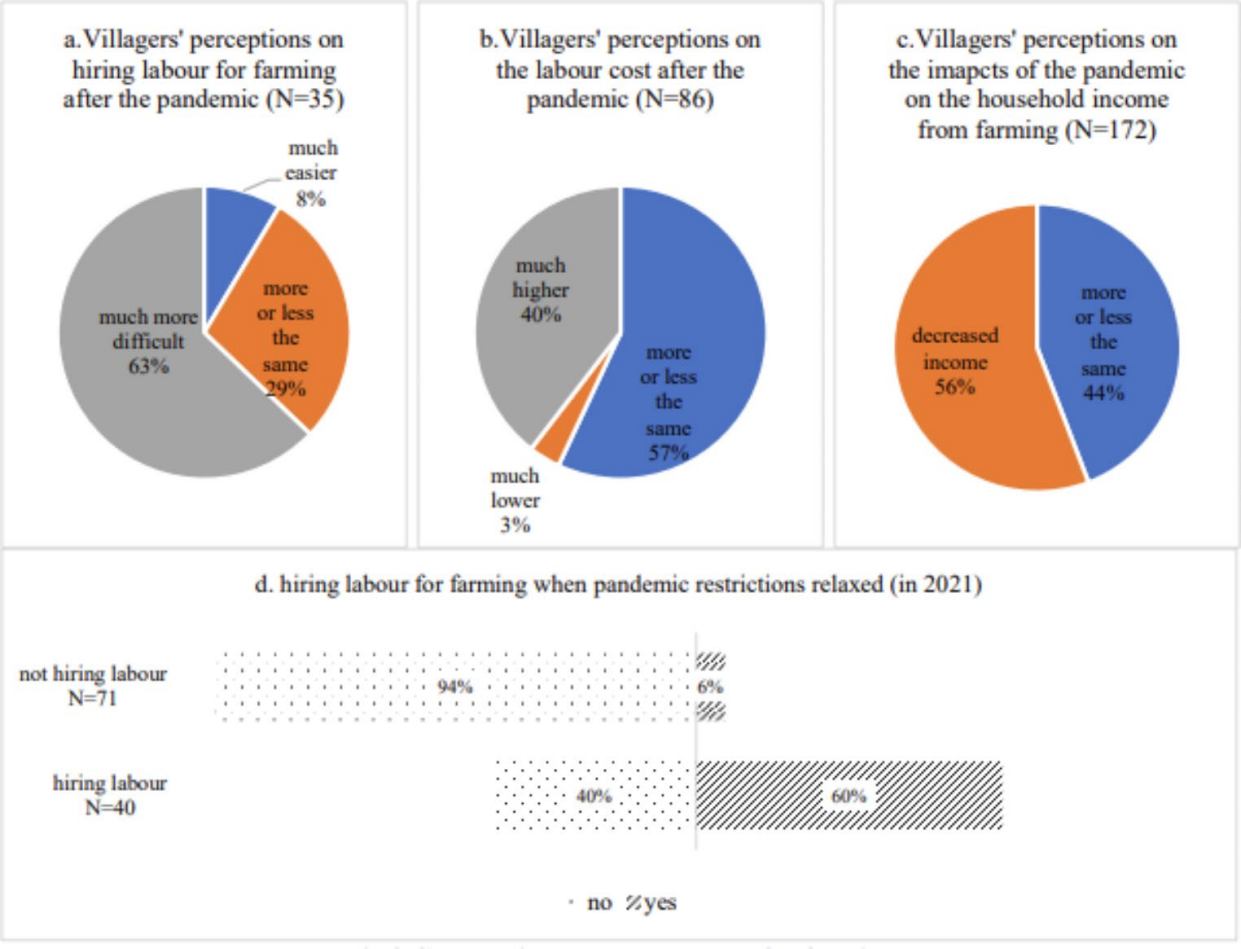
Changes in the labour supply for farming. Source: survey in ZK county in Yunnan province in August 2021. Note Only respondents who answered thre calculated.

Secondly, migrant workers from Myanmar could not enter China via both formal and informal ways^15^, as a wire mesh fence was set up by the Chinese government along its border.^16^ As a result, tens of thousands of migrants from neighbouring countries such as Myanmar, who used to migrate to southern China seasonally to undertake wage work (including cutting sugarcane), could not come to work in China. As mentioned above, more than 20,000 seasonal migrant workers from Myanmar worked in ZK County before 2019. This was no longer possible under the pandemic circumstances. Thus, the pandemic created a labour gap and significantly affected rural households that need to hire external farm labour^,^ as reflected in Fig. 9. Figure 9a shows that it became more difficult for many villagers to employ labour (63%), while for some (40%) labour costs became much higher (Fig. 9b). According to online interviews with villagers in 2021, labour costs for sugarcane cutting increased by 30 yuan per working hour in Yunnan province during the time of the pandemic, rising from 120 yuan per ton before the pandemic, to 150 yuan per ton by late 2023, and even higher on some days. It was also difficult to recruit enough labour during the pandemic. Labour that was available mainly comprised local workers, organized by the local government. In 2021, when the influence of the pandemic was less significant and many of the restrictions had been relaxed, 60% of households that used to hire labour started to do so again (Fig. 9d). However, many of those hired labourers were no longer from Myanmar but mainly from nearby regions due to the strict border controls. This meant increased costs for farming. As shown in Fig. 9c, around 56% of the villagers who responded to the survey felt that their income from farming has decreased.

The sudden and abrupt changes in labour supply were mediated by the state, in three specific ways. Firstly, to deal with the labour shortage, the county government set up a service team to coordinate the cutting, transporting and processing of sugarcane. It mobilized every villager, including those out-migrants who could not travel during the pandemic. The so-called “mutual help” team was not completely based on the principle of reciprocity under the moral economy. Rural households had to pay their neighbours/friends for the extra bundles cut by the mutual help team at the price of 2.5–3.0 yuan per bundle, which was much higher than the pay given to migrant Myanmar workers before the pandemic (1.5–1.8 yuan per bundle). This implies that the mutual help team was essentially an organized market and thus followed the capitalist logic. Secondly, in an attempt to support mechanized sugarcane production, the local government subsidized drone flying to spread crop pesticides and introduced the terracing projects. In ZK County, the county government and sugar companies invested 20 million yuan (10 million from each party) in converting 30,000 mu in 2021 (fieldnotes, April 20, 2021). Thirdly, the local government helped the villagers, particularly large-scale producers, to connect to markets. For instance, the county government reached out to the universities in Yunnan and Beijing to purchase macadamia nuts from Yunaoda Macadamia Corporation. Large farmers with contracts were thereby able to sell raw products to this corporation (fieldnotes, March 16, 2021).

However, the benefits from this state support during the pandemic were not evenly distributed. In most cases, middle and large producers got more support. The farming practices of smallholders were less likely to reach the scale needed to enable direct market access and to benefit from the organized local labour market and mechanization. Even for large producers, the support was not sufficient to cover their losses. As a large producer in MP Township complained, “It was getting inconvenient and inefficient to continue to use the mutual help team”. To solve the problem of labour shortage during the pandemic, he had to go to MD Township to hire workers, at a cost of 3 yuan per bundle. When it came to the end of the crushing season, the cost of labour became higher; the producer recalled that the highest price he paid was 4.5 yuan per bundle (fieldnotes, July 6, 2023).

Such changes in labour supply have significant impacts on the agrarian transformation in China’s agrarian system. However, the impacts were not discrete or isolated; rather, they were extensively shaped by the ongoing agrarian transformations in China. Moreover, it reflects the interconnected character of transformations.

### Differentiated impacts

**Fig. 10:**
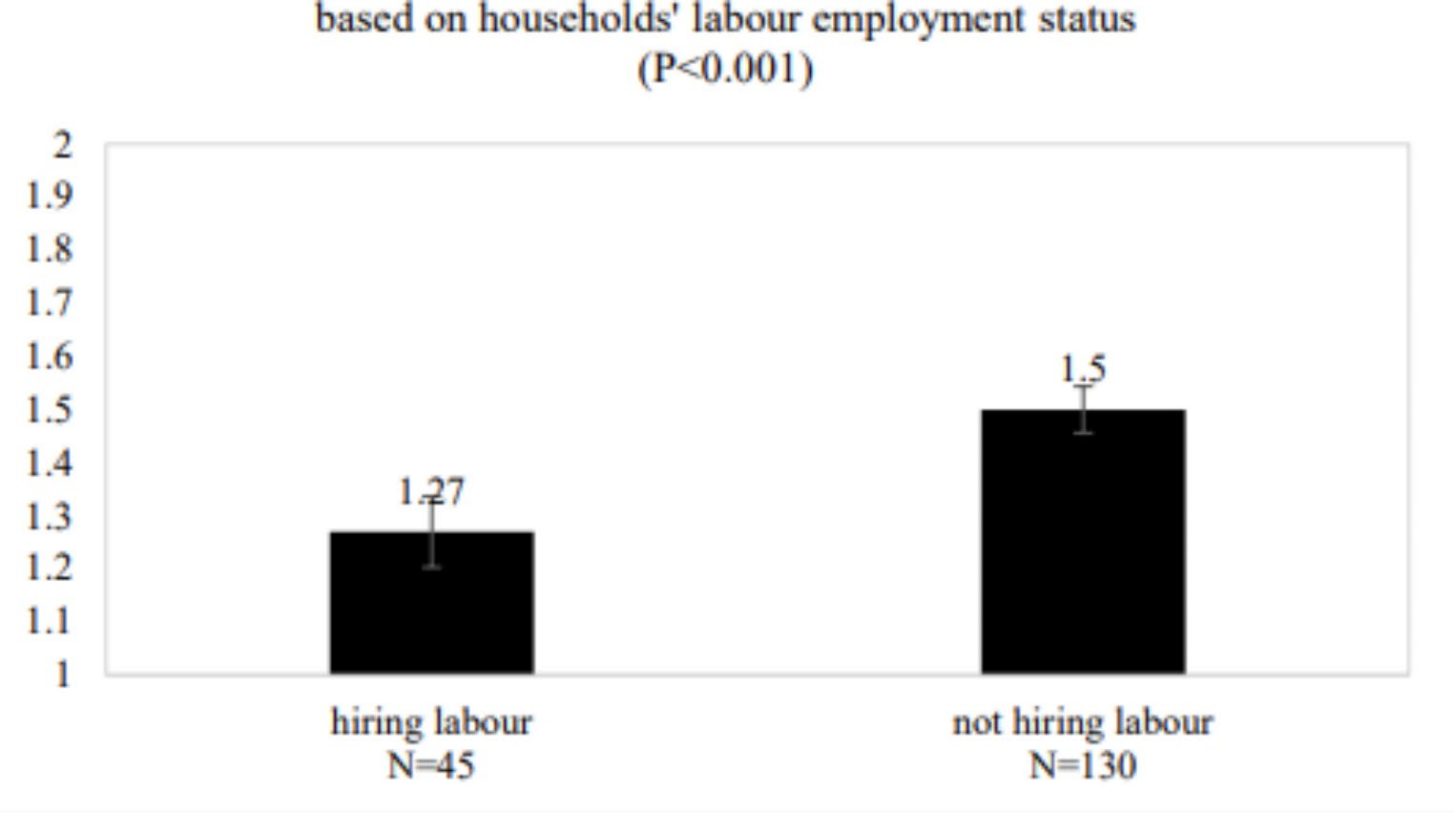
Households’ perspectives on the change of income from farming due to the impacts of Labour supply disruption. 1 = decreased income, 2 = more or less the same, 3 = increased income. So when the number is closer to 1, the negative impact on income is more significant. The significance of the differences is determined by the independent t-test. When *P* < 0.05, the difference among the groups is significant. More statistical results on this T-test, see Table 6 in the Appendix

The impacts of labour supply disruption were not homogeneous across the rural community; rather, they were highly uneven and closely associated with the social differentiation pattern prior to the outbreak of the pandemic, as reminded by Zhang and Hu 2021. As shown in Fig. 10, labour supply disruption had significantly different impacts on the income from farming of the households that do hire labour and those that do not. Compared with households that do not hire labour, households that hire labour felt more negatively affected by the pandemic. As mentioned above, those farms that did not hire labour can use family labour, particularly those who had been doing wage work in urban areas and were temporarily home due to the pandemic restrictions, according to our online interviews with township leaders conducted in March 2021. Thus, these farms were not affected by the increased labour costs resulting from labour supply changes, although the limits on the circulation of outputs still had negative impacts. This validates some enduring hypotheses about the resilience of smallholders who are less dependent on market shocks, such as disruption in the supply and cost of labour (van der Ploeg 2013).

**Fig. 11:**
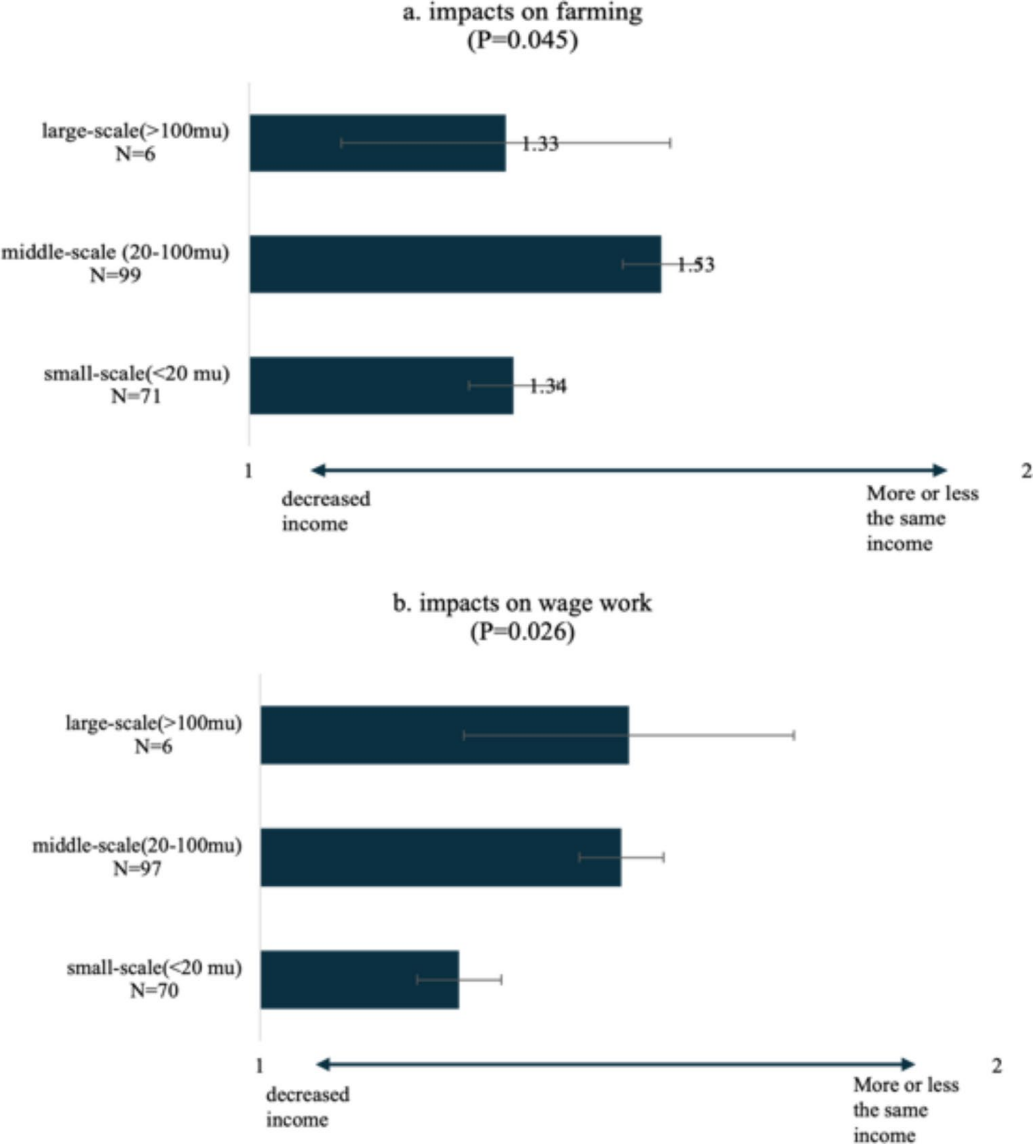
Households’ perspectives on change of income from farming and wage work due to the impact of changes in labour supply. Source: survey in ZK county in Yunnan province in August 2021. Note 1 = decreased income, 2 = more or less the same, 3 = increased income. The significance of the differences is determined by the ANOVA. When *P* < 0.05, the difference among the groups is significant. More statistical results on this ANOVA, see Table 7, 8 in the Appendix

Related to this, villagers with different sizes of landholdings tend to be affected differently. As shown in Fig. 11, there are significant differences among small-scale, middle-scale and large-scale farm owners regarding the pandemic’s impacts on farm income and wage income. In this study, small-scale farmers are defined as villagers whose land size is less than 20 mu, given that the median farmland size for villagers who do not hire labour for farming is 20 mu (see Table 3). Meanwhile, villagers who access more than 100 mu farmland are defined as *Dahu* (big landholders) in southern China; those who grow sugarcane usually get extra support from both the sugar companies and the local state (e.g. better access to loans and priorities in sugarcane cutting and transporting). Villagers who access more than 20 mu but less than 100 mu might hire labour but do not rely as heavily on external labour as those *Dahu*.

As shown in Fig. 11a, the farm income of villagers with middle-scale landholdings was the least negatively affected by labour supply disruption. This is because although these middle-class farm owners might normally (outside the period of the pandemic) have hired labour during the busy season (e.g., for sugarcane cutting), they could still maintain their farms by exploiting family labour for farming. Moreover, they were able to get extra help from family members who used to do migrant work but were unable to do so during the pandemic (Online interview, March 5, 2021). This is aligned with the textbook discussions of the persistence of family farms (see disuccsions above in Sect. 2).

In contrast, large-scale landholders felt their farming incomes were the most significantly affected. This is because they are highly dependent on employed labour for farming, and the labour gap cannot be filled by family labour given the scale of their sugarcane plantations (see Table 3 ). So, when cheap labourers from Myanmar were not available during the pandemic, their farming was inevitably affected.

For those households that access small-scale land plots, although their farming practices per se were hardly affected because they largely rely on family labour, their income from farming decreased. This is because smallholders are usually in a disadvantaged position in the market, and can thus become more vulnerable during a crisis. For instance, when selling sugarcane to sugar companies during the shortened harvest season caused by the pandemic^17^, smallholders usually had to wait behind large producers to get sugarcane tickets, which granted them permission to have their sugarcane cut and transported to mills, in accordance with the zoning system.^18^ As a result, there is an increase of sugarcane loss (rotted) when sugarcane was not harvested in time. Meanwhile, in order to sell other outputs (in addition to sugarcane), smallholders, particularly those in remote areas, are usually dependent on trade with brokers, but these informal arrangements were largely stopped due to the restrictions on movement during the pandemic.

As to the impacts on wage work, as shown in Fig. 10b, smallholders were more significantly affected than middle-scale and large-scale producers, because farming only provides a small share of their income while income from wage work is a more important livelihood source (also see Zhang 2015; van der Ploeg and Ye 2016). By contrast, large landholders’ income mainly relies on farming rather than wage work ( as reflected in Fig. 7). Thus, these large-scale producers perceived the least significant negative impact of the pandemic on income from wage work.

Thus, villagers were affected differently by the changes in the labour dynamics during the pandemic. Large-scale producers were the most affected. They encountered significant challenges in planting and harvesting due to serious labour shortages. Despite state support, their losses were difficult to offset because of their scale. Smallholders, while able to maintain their production by using the labourers left at home, struggled because of their unfavourable market position and limited state support. Middle-scale producers were able to benefit more from the state support on labour supply and were affected less significantly overall. These dynamics encapsulate the current state of Chinese agrarian transformation and reflect the role of land-related migrant labour flows in (re)shaping the transformation in rural Yunnan.

## Conclusion

From this research, we can clearly conclude that agrarian transformations in China are shaped not only by the processes and logic internal to China but also by dynamics in and associated with the agrarian transformation and politics in neighbouring countries. Given the extent of globalization, it would be difficult to completely separate the transformation of any region from global dynamics, as global capitalism and its accumulation processes and imperatives are interconnected — albeit in highly uneven ways — with the increased mobility of commodities, labour and capital (O’Connor 1998; Smith 2008). As this paper has shown, when many rural villagers take up off-farm work in nearby towns or further away in China, similarly large numbers of villagers from Myanmar go across the border to work in China. This cross-border labour migration to China is thus closely associated with the labour shortage created by the rural-urban migration within China. Meanwhile, in search of cheaper raw materials (land and labour), many Chinese companies and individuals are acquiring land outside China, particularly in Myanmar. These internal–external interactions all contribute to the transformation which is happening within China, and in turn (re)shape the transformation of Myanmar and beyond. In this sense, it is impossible to fully understand China’s agrarian transformation without exploring these internal–external dynamics across the Myanmar–China corridor, and vice versa.

In the case of southern China and adjacent countries, land and labour regimes are mutually embedded, and are necessarily multi-sited. In China, the rural labour shortage is related to the massive rural–urban migration and partly linked to the domestic rural land regime. Labour conditions in southern China are further changed by the large-scale inflow of cross-border migrants from Myanmar. These labour conditions shape the landscape in China.

Mutually embedded production and social reproduction processes are necessarily multi-sited within and across countries. For example, sugarcane production in China largely depends on the wage income gained from off-farm work in China and migrant workers from Myanmar. Similarly, the farming practices and social reproduction of the transnational migrant workers’ households in Myanmar are largely supported by remittances obtained from wage work in sugarcane plantations in China. Thus, interlinked through multi-sited land–labour dynamics, economic production and social reproduction are closely interconnected in southern China and Myanmar.

In closing, and as noted earlier, the scholarly literature on agrarian transformation in China is robust, shining a light on the land–labour nexus along the rural–urban continuum. Our contribution is a modest one: we have investigated a less-studied setting in which migrant farmers from across the border play an important role in agrarian transformation inside China. We argue that classic debates around social differentiation and the persistence of family farms amid generalized commodification, the rural–urban linkages that underpin capitalist development, and processes of capitalist penetration of the countryside, are increasingly in need of some revision to incorporate the important role played by cross-border migrant labour. In this context, we see the ability of family farms to persist and to hold on to their lands as being partly dependent on their ability to hire migrant wage workers. This applies not only in China; even in highly developed capitalist societies, such as in many parts of Europe, this is becoming a norm.

## Appendix

See Tables 4, 5, 6, 7, 8.

**Table 4:** T test result of land access between households who hire labour and who do not

	Mean	Median	Std. Deviation	95% Confidence Interval of the Difference		Sig.	t
				Lower	Upper		
Hiring labour (*N* = 46)	45.29	30	49.7	3.59	34.35	0.005	2.47
Not hiring labour (*N* = 130)	26.32	20	25.82				

**Table 5:** T test result of land access between households who do wage work and who do not

	N	Mean	Std. Deviation	95% Confidence Interval of the Difference		Sig.	t
				Lower	Upper		
No wage work	55	38.1664	48.9139	-3.91836	23.95538	0.002	1.44
Do wage work	121	28.1479	25.23587				

**Table 6:** T test result of changes in farming incomes between households who hire labour and who do not

	N	Mean	Std. Deviation	95% Confidence Interval of the Difference		Sig.	t
				Lower	Upper		
Hiring labour	45	1.27	0.447	-0.392	-0.074	< 0.001	-2.921
Not hiring labour	130	1.5	0.502				

**Table 7:** ANOVA result of changes in farming incomes among small-scale, middle-scale and large-scale holders

	N	Mean	Std. Deviation	Std. Error	95% Confidence Interval for Mean		Sig,
					Lower Bound	Upper Bound	
small-scale (< 20 mu)	71	1.34	0.476	0.057	1.23	1.45	0.045
middle-scale (20-100mu)	99	1.53	0.502	0.05	1.43	1.63	
large-scale (> 100mu)	6	1.33	0.516	0.211	0.79	1.88	

**Table 8:** ANOVA result of changes in wage incomes among small-scale, middle-scale and large-scale holders

	N	Mean	Std. Deviation	Std. Error	95% Confidence Interval for Mean		Sig,
					Lower Bound	Upper Bound	
small-scale(< 20 mu)	70	1.27	0.479	0.057	1.16	1.39	0.026
middle-scale(20-100mu)	97	1.49	0.561	0.057	1.38	1.61	
large-scale(> 100mu)	6	1.5	0.548	0.224	0.93	2.07	

## Footnotes

1 It is inspired by classic texts by Lenin (2004 [orig.1899]), Chayanov (1986 [orig.1925]) and Kautsky (1988 [orig.1899]) (for a synthetic discussion, see Akram-Lodhi and Kay 2010a; 2010b; Levien, Watts, and Yan 2018).

2 Source: Bureau of Statistics of Yunnan Province, http://stats.yn.gov.cn/tjsj/tjgb/202304/t20230407_1086700.html, accessed in March 2023.

3 For some exceptions, see e.g., Borras et al. 2018; Hua et al. 2019a; Hua, Zhang, and Kono 2022.

4 Hukou system is a formal registration of the population based on the household unit by the Chinese government. This system has a strict division between the rural and urban populations(Andreas and Zhan 2016; Chan and Zhang 1999).

5 According to Brookfield (2008*)*, the definition of family farms should consider the organisation of a farm. He argues that “[f]amily management, coupled with substantial work input, seems adequately to define family farms not only in Europe, but elsewhere.” (Brookfield 2008, p. 110).

6 For example, Xu 2019; Siciliano 2014; Andreas et al. 2020.

7 It is important to point out that a key context for the land and labour changing dynamics in the Myanmar-Yunnan corridor is the global land rush (Wolford et al. 2024), but we do not elaborate on this here.

8 In this paper, informal means of sugarcane production/informal land investments in Myanmar refer to land control in Myanmar for sugarcane production without formal contracts and a formal investment registration process.

9 Forestland in the southern China is undistributed, so the plots remain large. However, forestland has been controlled by various actors via distinct mechanisms including — with the rise of tree plantations — foreign companies (Xu 2020).

10 More dynamics on pin prick land deals, see Borras and Franco (2024*).*

11 Mu is a unit of area measurement. 15 mu equals to 1 ha.

12 But the actual scale of internal migration in this particualr region still requires further research.

13 More details on the mechanisms of land access by villagers, see Xu (2018*)*.

14 The policies were changed during and after the pandemic. More details on changes in the Yunnan province, see https://www.yn.gov.cn/ztgg/yqfk/yqztyqfkzccs/yqztsjzccs/, accessed on 11 September 2024.

15 The formal way of migration involves legal entry with an official visa, while the informal way refers to illegal migration, bypassing official channels.

16 See https://v.qq.com/x/page/p3259t07wze.html, and https://www.sohu.com/a/477931853_121124710. Accessed on 14 September 2023.

17 The harvest season was shortened to minimize gathering and prevent virus spread during the pandemic as part of the official measures.

18 For more details about the zoning system for sugarcane plantations in southern China, see Wang and Xu (2022).
